# Endogenous Sulfur Dioxide Improves the Survival Rate of Sepsis by Improving the Oxidative Stress Response during Lung Injury

**DOI:** 10.1155/2022/6339355

**Published:** 2022-02-27

**Authors:** Zhiwei Liu, Jiaqi Gao, Xin Ye, Cong Wang, Bin Zhao

**Affiliations:** ^1^Department of Emergency Medicine, Beijing Jishuitan Hospital, Beijing 100035, China; ^2^Department of Cardiology, Beijing Jishuitan Hospital, Beijing 100035, China

## Abstract

**Objective:**

To explore the regulation of endogenous sulfur dioxide on oxidative stress in lung injury induced by sepsis.

**Method:**

Forty male Sprague Dawley rats were divided into control, sepsis, sepsis + SO_2_, and SO_2_ group randomly used to observe survival rate. The other group of twenty-eight rats were randomly divided as the same manner for mechanism research. The number of WBCS and the percentage of PMN cells were calculated. The microphotographs of morphological changes and the index of quantitative assessment (IQA) of lung tissues were calculated. The ratio of wet/dry (W/D) of lung tissues was calculated. Levels of H_2_O_2_, MDA, NO, MPO, SOD, GSH-px, and TNF-*α* in plasma and lung tissues were measured.

**Result:**

The number of WBCS and the percentage of PMN cells decreased in sepsis (*p* all < 0.05), and rebound in sepsis+SO_2_ (*p* all < 0.05). The IQA and W/D of lung tissues increased in sepsis (*p* for *W*/*D* < 0.05), and decreased in sepsis+SO_2_ (*p* all < 0.05). H_2_O_2_ and MDA of plasma and lung tissues increased in sepsis (*p* all < 0.05) and rebound in sepsis+SO_2_ (*p* for H_2_O_2_ of plasma and lung tissues <0.05). NO and MPO of plasma and lung tissues increased in sepsis (*p* for NO and MPO of lung tissues <0.05) and rebound in sepsis+SO_2_ (*p* all < 0.05). SOD of plasma and lung tissues in sepsis group decreased (*p* all <0.05) and increased in sepsis+SO_2_ (*p* all < 0.05). GSH-px of plasma and lung tissues decreased in sepsis (*p* for plasma <0.05) and increased in sepsis+SO_2_ (*p* for GSH-px of lung tissues <0.05). TNF-*α* of plasma and lung tissues increased in sepsis (*p* all<0.05) and decreased in sepsis+SO_2_ (*p* for lung tissue <0.05).

**Conclusion:**

Endogenous sulfur dioxide improves the survival rate of sepsis by improving the oxidative stress response during lung injury.

## 1. Introduction

Sepsis could be induced by severe trauma, injury, shock, and major surgery. Sepsis may be developed to septic shock and multiple organ dysfunction. Sepsis is a common clinical disease and a common cause of death in patients. Lung injury is the most common complication and the most fatal complication [1]. Oxidative stress response is the key pathogenesis of lung injury induced by sepsis [2]. Endogenous SO_2_ acts as a protective role during the lung injury induced by ischemia-reperfusion [3]. Endogenous SO_2_ plays a protective role in acute lung injury caused by oleic acid by inhibiting oxidative stress [4]. However, whether endogenous sulfur dioxide can protect the lung injury of septic rats by improving the oxidative stress response, there is no relevant research at present. Therefore, this study puts forward the hypothesis that endogenous sulfur dioxide plays a protective role in the pathological process of sepsis by inhibiting the oxidative stress response of lung tissue during lung injury in sepsis. Therefore, this study took sepsis rats caused by cecal ligation and puncture to probe the regulation of endogenous sulfur dioxide about oxidative stress response in the process of septic lung injury.

## 2. Materials and Methods

### 2.1. Animal and Groups

This study is a randomized controlled animal experiment. This research proposal was approved by the ethics committee of Beijing Jishuitan Hospital. The ethics code of this research is Jilunke Shenzi No. 202006-26. A total of 68 Sprague-Dawley male rats at 8-week-old and weighed 200-250 g were bought from Animal Laboratory in the first hospital of Peking University. The sample size of the experiment depends on previous research [4].

#### 2.1.1. Rats Used to Observe Survival Rate

40 rats were used to observe the survival rate. They were randomly divided into four groups: control group, sepsis group, sepsis+SO_2_ group, and SO_2_ group. There were 10 rats in each group. After the completion of the operation, the 4 groups have all been observed for 72 hours, and the ratio of survival at 12, 24, 36, 48, and 72 hours after operation in rats was counted.

#### 2.1.2. Rats Allocated for Mechanism Research

The other groups of 28 male SD rats allocated for mechanism research were also randomly divided into four groups in the same manner, with four groups, i.e., control group, sepsis group, sepsis+SO_2_ group, and SO_2_ group. There were 7 rats in each group. The rats bled to death from abdominal aorta. These rats were anesthetized by 12% urethane 1 mL/kg intraperitoneally, after observation for 12 hours after the operation.

#### 2.1.3. Animal Model Preparation

For the rats in control group, the only treatment is opening the abdomen, turning the intestines, and then, closing the abdomen. The rats in SO_2_ group were treated with an intraperitoneal sulfur dioxide donor (Na_2_SO_3_/NaHSO_3_) of 0.5 mL/kg 30 minutes before the laparotomy was performed as an endogenous sulfur dioxide donor, and the rest of the operations were the same as those in control group. The Na_2_SO_3_/NaHSO_3_ solution was freshly prepared with physiological saline at 3 : 1 (0.54 : 0.18 mmol/kg). Rats in sepsis group were subjected to cecal ligation and perforation after laparotomy as follows [5]. The surgical operation method of cecal ligation and puncture was as follows: the animals were kept in an SPF environment and fasted 12 hours before the operation. After intraperitoneal injection of 13% pentobarbitone (1 mL/kg), they had experienced anesthetized fixation, shave, and alcohol disinfection. A 1 cm incision was made along the midline of the abdomen, the cecum exposed, and then ligated 1/2 of the cecum under the ileocecal valve. A 21-gauge needle is used to penetrate the cecum twice, and place a 3 mm wide rubber drainage strip through the cecum to prevent the needle hole from closing. After the cecum is admitted, the abdomen was closed and sutured layer by layer. The operation was strictly aseptic. Immediately after the surgery, 50 mL/kg normal saline was used for antishock. After the operation, the animals drank and ate freely. Rats in sepsis+SO_2_ group were injected sulfur dioxide donor (Na_2_SO_3_/NaHSO_3_) 0.5 mL/kg 30 minutes before the cecal ligation and perforation intraperitoneally. And the rest of the operations were the same as those in sepsis group. After observation time, the rats were bled to death from abdominal aorta with anesthetization by 12% urethane 1 mL/kg intraperitoneally.

### 2.2. Morphology of Lung Tissue under Light Microscope and Semiquantitative Assessment of Lung Injury (IQA)

The middle lobe of the right lung was stored, stained by HE, and observed by a 400x light microscope. Ten fields of view were taken from each slice for semiquantitative scoring of lung injury. Evaluation indicators included alveolar edema, inflammatory cell infiltration, and formation of the hyaline membrane. Each index was divided into four levels: normal, mild, moderate, and severe, which are scored as 0, 1, 2, and 3 correspondingly.

### 2.3. The Weight Ratio of Wet/Dry of Lung Tissue

The wet weight of the lung was recorded as soon as the lung was separated, and it was placed in 80°C incubator until the weight did not change and then recorded the dry weight. At last, the weight ratio of wet/dry was calculated.

### 2.4. Level of Oxidative Stress in Both Plasma and Lung Tissues

Levels of hydrogen peroxide (H_2_O_2_), malondialdehyde (MDA), nitric oxide (NO), and myeloperoxidase activity (MPO) in both plasma and lung tissues in each group were detected. Total glutathione oxidase (GSH-px) level and superoxide dismutase (SOD) activity in both plasma and lung tissues in each group were detected. All the above indicators were detected by ELISA.

### 2.5. Statistics

The data was statistically analyzed by using SPSS22.0 software. The data were recorded as mean ± standard deviation (mean ± SD). The comparison among the four groups was performed by one-way ANOVA, and *p* < 0.05 is considered as of statistical significance.

## 3. Results

### 3.1. The Survival Rate of Rats in Each Group

**Figure 1:**
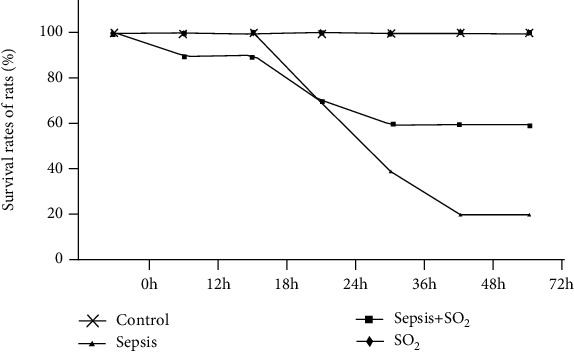
The survival rates of rats in each group.

The survival rates of rats in sepsis group were lower than the control group at 36 h, 48 h, and 72 h after the operation (*p* = 0.015, 0.001, and 0.001, respectively). The survival rates of rats in sepsis+ SO_2_ group were improved compared to the sepsis group at 12 h, 24 h, 36 h, 48 h, and 72 h after the operation, but the difference were not statistically significant (*p* = 1.000, 1.000, 0.371, 0.171, and 0.171, respectively, Figure 1, Table 1).

### 3.2. The WBC and Neutrophil of Rats in Each Group

**Figure 2:**
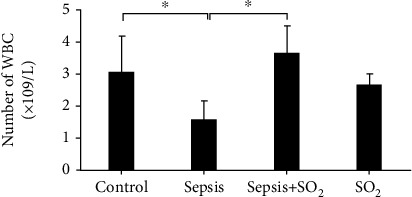
Number of WBCS in each group (×10^9^/L). ^∗^*p* < 0.05.

**Figure 3:**
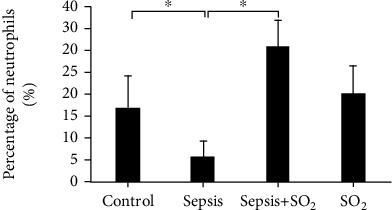
Percentage of neutrophils in each group (%). ^∗^*p* < 0.05.

The blood WBC of rats in control group, sepsis group, sepsis+SO_2_ group, and SO_2_ group were 3.07 ± 1.074 × 10^9^/L, 1.61 ± 0.538 × 10^9^/L, 3.66 ± 0.790 × 10^9^/L, and 2.69 ± 0.278 × 10^9^/L, respectively. The white blood cell count of rats in sepsis group was lower than the control group (*p* = 0.001). The white blood cell count of rats in sepsis+SO_2_ group was higher than the sepsis group (*p* = 0.000, Figure 2). The percentages of neutrophils in the blood of rats in control group, sepsis group, sepsis+SO_2_ group, and SO_2_ group were 17.81 ± 7.34%, 4.40 ± 1.66%, 31.0 ± 5.85%, and 20.5 ± 5.85%, respectively. The percentage of blood neutrophils in sepsis group was lower than the control group (*p* = 0.000). Compared with sepsis group, the percentage of blood neutrophils in sepsis+SO_2_ group was higher (*p* = 0.000, Figure 3).

### 3.3. Morphology of Lung Tissue in Rats of Each Group under Light Microscope

**Figure 4:**
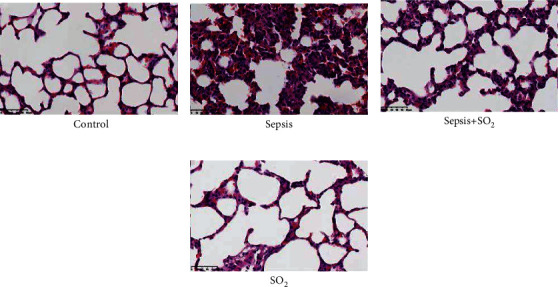
Microphotographs of morphological change of lung tissues (×40). Hematoxylin and eosin staining: (a) control, (b) sepsis, (c) sepsis+SO_2_, and (d) SO_2_.

Under the light microscope, the lung tissue in control group was roughly normal, the alveolar walls were intact, no obvious secretions were seen in the alveolar cavity, and there was no inflammatory cell infiltration and red blood cell exudation. In sepsis group, changes in lung tissue occurred. The alveolar structure was incomplete. The alveolar compartment was thickened. As for lung interstitium and alveolar edema, compared with sepsis group, the alveolar structure of sepsis+SO_2_ group was more complete, the alveolar interval narrowed, and the interstitial edema was reduced (Figure 4).

### 3.4. IQA of Lung Tissue in Each Group

**Figure 5:**
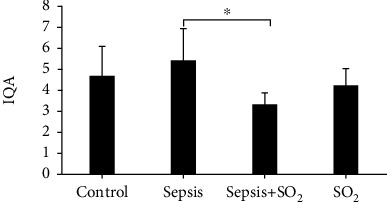
IQA of lung tissue in each group. ^∗^*p* < 0.05.

IQA of lung tissue in control group, sepsis group, sepsis+SO_2_ group, and SO_2_ group was 4.71 ± 1.380, 5.43 ± 1.512, 3.33 ± 0.516, and 4.28 ± 0.756, respectively. IQA of lung injury in sepsis group was increased compared with control group (*p* = 0.253). While compared with sepsis group, IQA of lung injury in sepsis+SO_2_ group was reduced (*p* = 0.003, Figure 5).

### 3.5. Ratios of *W*/*D* of Lung Tissue in Each Group

**Figure 6:**
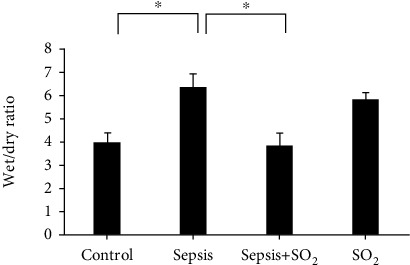
The ratios of *W*/*D* of lung tissue in each group. ^∗^*p* < 0.05.

Ratios of *W*/*D* of lung tissue in control group, sepsis group, sepsis+SO_2_ group, and SO_2_ group were 3.90 ± 0.334, 6.32 ± 0.393, 3.82 ± 0.514, and 5.83 ± 0.282, respectively. The *W*/*D* of lung tissue in sepsis group increased compared with control group (*p* = 0.000). The *W*/*D* of lung tissue in sepsis+SO_2_ group was lower compared with sepsis group (*p* = 0.000, Figure 6).

### 3.6. Oxidation System Indicators in Plasma and Lung Tissues in Each Group

#### 3.6.1. Levels of H_2_O_2_ in Plasma and Lung Tissues in Each Group

**Figure 7:**
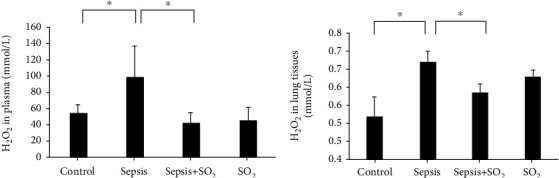
Levels of H_2_O_2_ in plasma and lung tissues in each group (mmol/L). (a) Plasma; (b) lung tissues. ^∗^*p* < 0.05.

Levels of H_2_O_2_ in plasma in control group, sepsis group, sepsis+SO_2_ group, and SO_2_ group were 53.8 ± 10.1 mmol/L, 98.4 ± 37.4 mmol/L, 43.1 ± 9.22 mmol/L, and 44.7 ± 14.0 mmol/L, respectively. Levels of H_2_O_2_ in lung tissues in control group, sepsis group, sepsis+SO_2_ group, and SO_2_ group were 0.513 ± 0.0602 mmol/g, 0.671 ± 0.0307 mmol/g, 0.586 ± 0.0227 mmol/g, and 0.630 ± 0.0180 mmol/g, respectively. Levels of H_2_O_2_ in plasma and lung tissues in sepsis group increased compared with the control group (*p* = 0.001, *p* = 0.000, respectively). Levels of H_2_O_2_ in plasma and lung tissues in sepsis+SO_2_ group were reduced compared with the sepsis group (*p* = 0.000, *p* = 0.000, respectively, Figure 7).

#### 3.6.2. Levels of MDA in Plasma and Lung Tissues in Each Group

**Figure 8:**
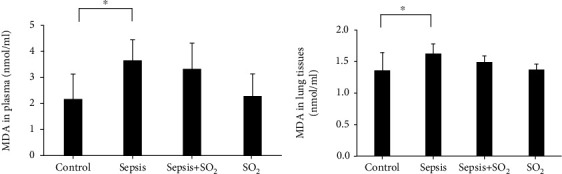
Levels of MDA in plasma and lung tissues in each group (nmol/mL). (a) Plasma; (b) lung tissues. ^∗^*p* < 0.05.

Levels of MDA in plasma in control group, sepsis group, sepsis+SO_2_ group, and SO_2_ group were 2.17 ± 0.898 nmol/mL, 3.63 ± 0.745 nmol/mL, 3.27 ± 0.969 nmol/mL, and 2.29 ± 0.831 nmol/mL, respectively. Levels of MDA in lung tissues in control group, sepsis group, sepsis+SO_2_ group, and SO_2_ group were 1.35 ± 0.2732 nmol/mg, 1.62 ± 0.1445 nmol/mg, 1.48 ± 0.0885 nmol/mg, and 1.36 ± 0.0843 nmol/mg, respectively. Compared with control group, levels of MDA in plasma and lung tissues in sepsis group increased (*p* = 0.004, *p* = 0.007, respectively). Compared with sepsis group, levels of MDA in plasma and lung tissues in sepsis+SO_2_ group also showed a decreasing trend (*p* = 0.450, *p* = 0.000, respectively, Figure 8).

#### 3.6.3. Levels of NO in Plasma and Lung Tissues in Each Group

**Figure 9:**
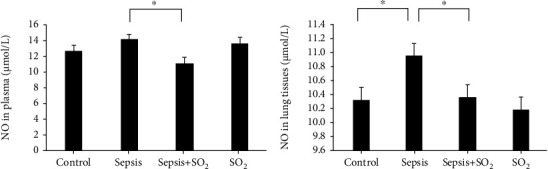
Levels of NO in plasma and lung tissues in each group (*μ*mol/L). (a) Plasma; (b) lung tissues. ^∗^*p* < 0.05.

Levels of NO in plasma in control group, sepsis group, sepsis+SO_2_ group, and SO_2_ group were 12.62 ± 1.34 *μ*mol/L, 14.10 ± 1.42 *μ*mol/L, 11.12 ± 1.11 *μ*mol/L, and 13.68 ± 2.32 *μ*mol/L, respectively. Levels of NO in lung tissues in control group, sepsis group, sepsis+SO_2_ group, and SO_2_ group were 10.3 ± 0.894 mmol/g, 11.0 ± 0.360 mmol/g, 10.4 ± 0.327 mmol/g, and 10.2 ± 0.152 mmol/g, respectively. Levels of NO in plasma and lung tissues in sepsis group also showed an increasing trend compared with the control group (*p* = 0.100, *p* = 0.032, respectively). Compared with sepsis group, levels of NO in plasma and lung tissues in sepsis+SO_2_ group were lower (*p* = 0.002, *p* = 0.049, respectively, Figure 9).

#### 3.6.4. Levels of MPO in Plasma and Lung Tissues in Each Group

**Figure 10:**
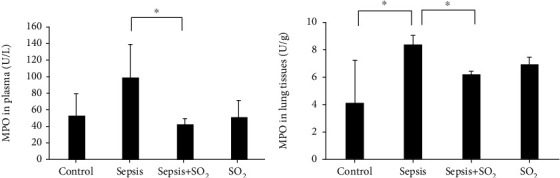
Levels of MPO in plasma and lung tissues in each group (U/L). (a) Plasma; (b) lung tissues. ^∗^*p* < 0.05.

Levels of MPO in plasma in control group, sepsis group, sepsis+SO_2_ group, and SO_2_ group were 51.8 ± 25.5 U/L, 85.1 ± 52.2 U/L, 38.1 ± 9.89 U/L, and 50.3 ± 19.5 U/L, respectively. Levels of MPO in lung tissues in control group, sepsis group, sepsis+SO_2_ group, and SO_2_ group were 4.05 ± 3.084 U/g, 8.26 ± 0.637 U/g, 6.11 ± 0.195 U/g, and 6.88 ± 0.444 U/g, respectively. Compared with control group, levels of MPO in plasma and lung tissues in sepsis group showed an increasing trend (*p* = 0.056, *p* = 0.000, respectively). Compared with sepsis group, MPO level in plasma and lung tissues in sepsis+SO_2_ group were significantly lower (*p* = 0.009, *p* = 0.027, respectively, Figure 10).

### 3.7. Antioxidant System Indicators in Plasma and Lung Tissues in Each Group

#### 3.7.1. Levels of SOD in Plasma and Lung Tissues in Each Group

**Figure 11:**
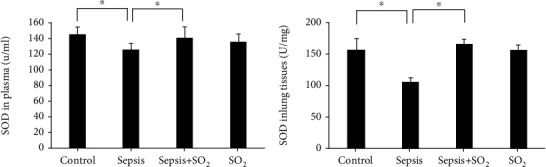
Levels of SOD in plasma and lung tissues in each group (U/mL). (a) Plasma; (b) lung tissues. ^∗^*p* < 0.05.

Levels of SOD in plasma in control group, sepsis group, sepsis+SO_2_ group, and SO_2_ group were 143 ± 9.45 U/mL, 124 ± 6.46 U/mL, 138 ± 14.22 U/mL, and 134 ± 8.91 U/mL, respectively. Levels of SOD in lung tissues in control group, sepsis group, sepsis+SO_2_ group, and SO_2_ group were 156.0 ± 17.70 U/mg, 106.2 ± 5.25 U/mg, 165.9 ± 6.46 U/mg, and 157.0 ± 7.13 U/mg, respectively. Levels of SOD level in plasma and lung tissues in sepsis group decreased compared with control group (*p* = 0.003, *p* = 0.001, respectively). SOD level in plasma and lung tissues in sepsis+SO_2_ group increased compared with sepsis group (*p* = 0.021, *p* = 0.000, respectively, Figure 11).

#### 3.7.2. Levels of GSH-Px in Plasma and Lung Tissues in Each Group

**Figure 12:**
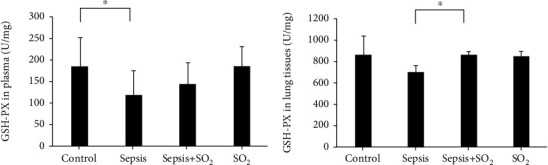
Levels of GSH-px in plasma and lung tissues in each group (U/mL). (a) Plasma; (b) lung tissues. ^∗^*p* < 0.05.

Levels of GSH-px in plasma in control group, sepsis group, sepsis+SO_2_ group, and SO_2_ group were 186 ± 64.7 U/mL, 118 ± 56.2 U/mL, 147 ± 47.2 U/mL, and 186 ± 44.6 U/mL, respectively. Levels of GSH-px in lung tissues in control group, sepsis group, sepsis+SO_2_ group, and SO_2_ group were 41.1 ± 7.33 U/mg, 51.2 ± 1.92 U/mg, 45.0 ± 5.13 U/mg, and 44.2 ± 4.30 U/mg, respectively. Compared with control group, levels of GSH-px in plasma and lung tissues in sepsis group decreased (*p* = 0.027, *p* = 0.295, respectively). Compared with sepsis group, levels of GSH-px in sepsis+SO_2_ group also showed an increasing trend (*p* = 0.333, *p* = 0.001, respectively, Figure 12).

### 3.8. Levels of TNF-*α* in Plasma and Lung Tissues in Each Group

**Figure 13:**
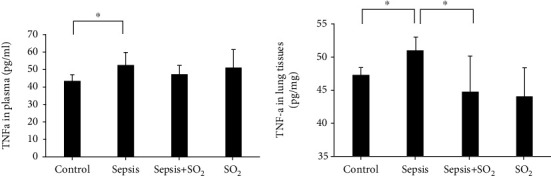
Levels of TNF-*α* in plasma and lung tissues in each group. (a) Plasma; (b) lung tissues. ^∗^*p* < 0.05.

Levels of TNF-*α* in plasma and lung tissues in control group, sepsis group, sepsis+SO_2_ group, and SO_2_ group were 43.8 ± 2.94 pg/mL, 52.2 ± 7.50 pg/mL, 47.6 ± 4.59 pg/mL, and 50.8 ± 10.60 pg/mL, respectively. Levels of TNF-*α* in lung tissues in control group, sepsis group, sepsis+SO_2_ group, and SO_2_ group were 41.1 ± 7.33 pg/mg, 48.5 ± 2.80 pg/mg, 45.0 ± 5.13 pg/mg, and 44.2 ± 4.30 pg/mg, respectively. Compared with control group, levels of TNF-*α* in plasma and lung tissues in sepsis group increased (*p* = 0.035, *p* = 0.001, respectively). Compared with sepsis group, levels of TNF-*α* in sepsis+SO_2_ group also showed an decreasing trend (*p* = 0.236, *p* = 0.040, respectively, Figure 13).

## 4. Discussion

Sepsis is a common and critical complication, which could be induced by severe trauma, injury, shock, or major surgery. Sepsis may develop into septic shock. Though clinical technology develops fast, the high morbidity and mortality of sepsis is still a major problem [6, 7]. Previous studies have shown that septic lung injury can lead to the interaction of cAMP and various hormone levels, resulting in changes in lung tissue structure [8–12]. In this study, the survival rate of 40 SD rats was studied through the CLP sepsis model. The result suggested that the survival rate of septic rats decreased after 24 hours, and the final survival rate was only 20%. Li et al. found that the mortality rate of sepsis rat models began to decrease from 24 hours after the operation and finally reached 30%, which was consistent with the result of this research [13]. The result suggested that the survival rate in sepsis group was reduced. The survival rate was increased to 60% after the intervention of sulfur dioxide. The results demonstrated that sulfur dioxide can increase the survival rate of sepsis rates and reduce the mortality rate during the onset of sepsis. At the same time, this study established a CLP model in 28 rats, and the number of WBC and the percentage of neutrophils in the sepsis group were reduced. This result has the same means as that reported by Wang LY, suggesting that the CLP sepsis model in this study was successfully made [13–15].

Lung tissue is the common easily affected organ in sepsis, causing septic lung injury [16]. Septic lung injury is an important complication of sepsis, with the highest death ratio, and it is also an important reason for death from sepsis [17, 18]. In this study, after cecal ligation and perforation were given to male rats, under a light microscope, the following could be observed: the lung tissue structure was incomplete, lung capillary permeability increased, alveolar septum thickened, lung interstitium increased, and alveolar edema occurred. The semiquantitative score of lung tissue increased. Deng et al. also found the increase of IQA of lung tissues [14]. The *W*/*D* ratio of lung tissue increased. These changes are consistent with the pathological process of acute lung injury and once again confirmed that sepsis can be caused by acute lung injury. Endogenous sulfur dioxide may play a protective role in lung injury as found in previous studies [19]. In this study, after the endogenous SO_2_ donor Na_2_SO_3_/NaHSO_3_ was given, the lung tissue structure, lung capillary permeability, and the obvious thickening of the alveolar compartment were all improved; the wet weight/dry weight ratio of lung tissue and the histology of lung injury was improved. The IQA were lower than before, also confirming that SO_2_ is a protective role during lung injury induced by sepsis.

The imbalance between oxidation and antioxidants in the oxidative stress response is a major mechanism during septic lung injury and chronic obstructive pulmonary disease [20]. This study also found that the oxidation system indicators of the oxidative stress response in sepsis group including H_2_O_2_, MDA, NO level, and MPO activity increased compared with the control group. This result is the same as the results of Rasooli et al. [21], antioxidant system indicators including SOD activity and GSH-px level are reduced, and the above results are consistent with previous research results [22–24], once again confirming that oxidation and antioxidant imbalance take part in the pathogenesis of septic lung injury. Other studies have also confirmed that sulfur dioxide could regulate the oxidative stress response [25, 26]. In this study, it was found that after sulfur dioxide intervention, the oxidation system indicators H_2_O_2_, MDA, NO level, and MPO activity decreased. The antioxidant system indicators including SOD activity and GSH-px level raised. The results show that sulfur dioxide can protect lung tissue by improving the oxidation and antioxidant effects of sepsis.

In the pathological process of sepsis, TNF-*α* can induce the release of downstream inflammatory mediators to cause oxidative stress imbalance. At the same time, our research found that the content of TNF-*α* is significantly increased during lung injury. In the sepsis+SO_2_ group, levels of TNF-*α* in plasma and lung tissue were reduced, and the oxidative stress index was significantly improved compared with the sepsis group. These results suggest that the administration of SO_2_ can inhibit the expression of TNF-*α* in the case of sepsis lung injury. Inhibit the oxidative stress response, thereby playing a protective role in septic lung injury.

In summary, sulfur dioxide can improve the survival rate of sepsis by reducing lung injury caused by sepsis, and its mechanism is related to inhibition of the oxidation effect induced by sepsis during lung injury and improving antioxidant effect. This discovery deepens the understanding of sepsis in clinical work and provides new ideas for the treatment of sepsis and the development of new drugs. However, this study did not explore more specific mechanisms. These specific mechanisms need to be carried out in future research.

## Figures and Tables

**Table 1:** The survival rates of rats in each group.

After CLP		Group		*χ* ^2^	*p*	Group		*χ* ^2^	*p*
		Sepsis	Control			Sepsis	Sepsis+SO_2_		
12 h	Death	0	0			0	1		1.000
	Survival	10	10			10	9		
24 h	Death	3	0	1.569	.210	3	3	0.000	1.000
	Survival	7	10			7	7		
36 h	Death	6	0	5.952	.015	6	4	.800^a^	.371
	Survival	4	10			4	6		
48 h	Death	8	0	10.208	.001	8	4	1.875	.171
	Survival	2	10			2	6		
72 h	Death	8	0	10.208	.001	8	4	1.875	.171
	Survival	2	10			2	6		

## Data Availability

The data used to support the findings of this study are included within the article.
